# Through-Plane and In-Plane Thermal Diffusivity Determination of Graphene Nanoplatelets by Photothermal Beam Deflection Spectrometry

**DOI:** 10.3390/ma14237273

**Published:** 2021-11-28

**Authors:** Humberto Cabrera, Dorota Korte, Hanna Budasheva, Behnaz Abbasgholi N. Asbaghi, Stefano Bellucci

**Affiliations:** 1 Optics Lab, STI Unit, The Abdus Salam International Centre for Theoretical Physics, Strada Costiera 11, 34151 Trieste, Italy; hcabrera@ictp.it (H.C.); be.asbaghi@yahoo.com (B.A.N.A.); 2 Laboratory for Environmental and Life Sciences, University of Nova Gorica, Vipavska 13, SI-5000 Nova Gorica, Slovenia; dorota.korte@ung.si (D.K.); hanna.budasheva@ung.si (H.B.); 3 Laboratori Nazionali di Frascati, Istituto Nazionale di Fisica Nucleare, Via E. Fermi 54, 00044 Frascati, Italy

**Keywords:** graphene nanoplatelets, thermal diffusivity, thermal conductivity, photothermal spectrometry

## Abstract

In this work, in-plane and through-plane thermal diffusivities and conductivities of a freestanding sheet of graphene nanoplatelets are determined using photothermal beam deflection spectrometry. Two experimental methods were employed in order to observe the effect of load pressures on the thermal diffusivity and conductivity of the materials. The in-plane thermal diffusivity was determined by the use of a slope method supported by a new theoretical model, whereas the through-plane thermal diffusivity was determined by a frequency scan method in which the obtained data were processed with a specifically developed least-squares data processing algorithm. On the basis of the determined values, the in-plane and through-plane thermal conductivities and their dependences on the values of thermal diffusivity were found. The results show a significant difference in the character of thermal parameter dependence between the two methods. In the case of the in-plane configuration of the experimental setup, the thermal conductivity decreases with the increase in thermal diffusivity, whereas with the through-plane variant, the thermal conductivity increases with an increase in thermal diffusivity for the whole range of the loading pressure used. This behavior is due to the dependence of heat propagation on changes introduced in the graphene nano-platelets structure by compression.

## 1. Introduction

Graphene is a 2D layer of carbon atoms arranged in hexagonal rings. Currently, it attracts a growing research interest due to its unique properties, including structure (the theoretical value of the specific surface area is 2630 m^2^ g^−1^), rigidity (Young’s modulus is approximately 1100 GPa) and strength (the fracture strength is about 125 GPa). Furthermore, it has a very high electrical current density (at the level of 108 Acm^−2^) and mobility of charge carriers (2 × 10^5^ cm^2^V^−1^s^−1^) [1,2,3,4]. Its thermal conductivity depends on the direction of heat propagation and reaches a value of 3000 Wm^−1^K^−1^ in the parallel direction and 5 Wm^−1^K^−1^ in the perpendicular direction to the sample surface [5,6]. Moreover, the thermal diffusivity of graphene or freestanding graphene depends on the number of layers and can reach a value of 6.5 × 10^−4^ m^2^ s^−1^ [7]. Graphene is also chemically stable and does not react with other substances [1,2]. However, it can be functionalized by various atoms, nanoparticle composites and chemical groups to produce different graphene-based hybrids and composites, which thus inherit further its performance, to be applied in medicine, energy conversion, as sensors, storage devices, structural materials, reinforced composites and catalysts in the process of water purification [8,9].

Graphene nanoplatelets (GNP) consist of a few graphene layers prepared by the exfoliation method. Such GNP material has found many applications in different fields of industry, science and medicine; thus, its characterization is crucial for predicting its behavior in practical uses [10,11]. Thermal properties are of high importance since they are widely applied as heat conductors and heat energy storage systems. Although thermal properties have already been investigated, e.g., through-plane thermal diffusivity and conductivity, data for the in-plane values are still missing [12,13]. Moreover, these measurements were performed using traditional methods such as laser flash, which cannot measure the in-plane properties. Additionally, complementary independent measurement with a highly sensitive method is required to confirm the reliability of the previous measurement.

Photothermal beam deflection spectrometry (BDS) is nowadays widely used for materials characterization to determine their thermal, optical, elastic and related properties (structural, transport) [14,15,16,17]. It is a non-contact and non-destructive technique that provides high spectral, spatial, and temporal resolution, as well as high sensitivity. This is achieved as a result of indirect measurement of physical phenomenon induced in the inner part of the examined sample, its surface and/or its surroundings caused by a heat source. Thus, there is no direct contact between the sample and the detector, which enables to avoid the mechanical interference and noises as well as provides a non-destructive way of measurement. BDS belongs to a group of photothermal techniques [18,19,20,21,22] in which the examined sample is irradiated by a modulated excitation laser beam. The absorbed photon energy is converted into heat, inducing collective vibration of the sample’s phonons and/or electrons. The heat transfer causes temperature oscillations (TOs) in the sample and further in an adjacent gas layer close to its surface. The generation of TOs leads to the creation of a density gradient in the adjacent gas layer and a change in its index of refraction. These changes are detected by a probe laser beam (PB) skimming the sample surface, suffering a deflection that causes phase and amplitude changes related to the beam position changes at the detector. BDS can be applied in the case of both isotropic and anisotropic materials, solids and thin film, semiconductors, composites and complex materials, etc. [23,24].

In this study, the beam deflection spectrometry was used to determine the in-plane and through-plane thermal diffusivity of GNP to which different pressing loads were applied, which enables predicting their possible application as heat conductors or in heat storage systems. For that purpose, a new theoretical description based on complex geometrical optics equations was developed and applied for determining the GNP in-plane and through-plane thermal properties.

## 2. Photothermal Beam Deflection Spectrometry Theory

### 2.1. Surface Scan Method

In order to determine TOs in the fluid over the GNP sample surface, it is necessary to solve the heat diffusion equation:

(1) $$∆\upsilon \left(y,z,t\right)-\frac{1}{D_{Ti}}\frac{\partial \upsilon \left(y,z,t\right)}{\partial t}=-\frac{q}{\kappa_{Ti}}$$

where *D*_Ti_ and *K*_Ti_ are the thermal diffusivity and conductivity of a material or a fluid, respectively. Since GNP samples are strongly absorbing, it can be assumed that the EB absorbance occurs at the surface of the sample, and they do not occur any internal heat sources (*q* = 0).

The real part of the solution of Equation (1) with the boundary condition of temperature and flux of heat continuity at the borders of sample-surrounding fluid is given by:

(2) $$\upsilon_{f}\left(y,z,t\right)=\sqrt{\psi_{fR}^{2}\left(y,z\right)+\psi_{fI}^{2}\left(y,z\right)}\cos\left(\omega t+\mathrm{atan}\frac{\psi_{fI}\left(y,z\right)}{\psi_{fR}\left(y,z\right)}\right)$$

where $\psi_{fR}\left(y,z\right)$ and $\psi_{fI}\left(y,z\right)$ are the real and imaginary part of the temperature distribution in the fluid above the sample, and ω is the angular modulation frequency of EB (see Appendix A).

In the presented study, the interaction of PB with TOs is performed by the use of complex ray theory (CRT) [24]. According to CRT, the PB is a bundle of rays propagating in a complex space which coordinates are found on the grounds of rays’ equations that in an optically homogenous medium are of the form:

(3) $$x(\tau )=n_{0}\tau \sqrt{1+\frac{\xi^{2}+\eta^{2}}{z_{RC}^{2}}}y(\tau )=\eta \left(1+i\frac{n_{0}\tau }{z_{RC}}\right)z(\tau )=\xi \left(1+i\frac{n_{0}\tau }{z_{RC}}\right)$$

where *ξ* and *η* are the coordinates of the ray in the input plane of the experimental setup (*z* = 0), τ is the running coordinate along the ray trajectory, *z_RC_ = z_R_–iL*_0_ is the complex Rayleigh’s length, *z_R_* = *ka*^2^*n*_0_ is the Rayleigh’s length, *L*_0_ is the focal distance, *a* is the radius of the probe beam in its waist and *n*_0_ is the index of refraction in ambient temperature, whereas *k* = 2*π/λ* is the wave number, and λ is the wavelength of the probe beam in vacuum.

The TOs cause change in the refractive index, as well as its gradient in the optically heated region. The PB interaction with TOs results in its deflection on the thermal gradient, which is reflected in the change in PB’s trajectory; this means the corrections *y*_1_ and *z*_1_ to its coordinates expressed by Equation (4) must be found (see Appendix B):

(4) $$x_{1}\left(\xi ,\eta ,\tau \right)=n_{0}^{2}s_{T}\overset{\tau }{\underset{0}{\int }}\left(\tau -\tau^{\prime }\right)\frac{\partial \upsilon_{f}}{\partial x}d\tau^{\prime }$$

(5) $$y_{1}\left(\xi ,\eta ,\tau \right)=n_{0}^{2}s_{T}\overset{\tau }{\underset{0}{\int }}\left(\tau -\tau^{\prime }\right)\frac{\partial \upsilon_{f}}{\partial y}d\tau^{\prime }$$

where *n*_0_ is the refractive index of undisturbed fluid; *s_T_* = (1/*n*_0_)(*dn/dT*) is the thermal sensitivity, and *dn/dT* is the temperature coefficient of refractive index; *τ* is the running complex coordinate along the PB trajectory; *ξ* and *η* are the PB’s coordinates in the input plane of the experimental setup (*z* = 0).

The change in the fluid’s refractive index and its gradient, caused by TOs, leads to the change in PB’s optical path and finally to the change in the PB phase (see Appendix C):

(6) $$\Phi_{1}=kn_{0}^{2}s_{T}\int_{0}^{\tau }\vartheta_{f}\left[z\left(\tau^{\prime }\right)\right]d\tau^{\prime }.$$

The deflection of PB causes the change in PB divergence and, finally, the amplitude of its electric field according to the formula (see Appendix D):

(7) $$A\left(\tau \right)=E_{0}\frac{z_{R}}{z_{Rc}}{\left[\frac{D\left(0\right)}{D\left(\tau \right)}\right]}^{1/2}$$

where *D*(0) and *D*(*τ*) are the transformation Jacobian of the rays’ coordinates from the input plane of the experimental setup along the trajectory of the rays, *E*_0_ is the amplitude of the electric field of undisturbed PB in its waist.

At the detector plane (*z_D_*), the amplitude of the PB electric field has the form (see Appendix D):

(8) $$A\left(z_{D}\right)=A_{0}\left[1+a_{1}\left(z_{D}\right)\right]$$

where $A_{0}=E_{0}z_{R}/z_{RC}$ and $a_{1}\left(z_{D}\right)$ are the corrections to the amplitude resulting from its deflection on TOs gradients.

The PB intensity changes caused by its interaction with TOs at the detector is proportional to the squared value of the PB electric field distribution U(*x_D_*, *y_D_*, *z_D_*) and can be written as:

(9) $$I\left(x_{D},y_{D},z_{D}\right)∼{\left|U\left(x_{D},y_{D},z_{D}\right)\right|}^{2}=I_{0g}\left(x_{D},y_{D},z_{D}\right)\left[1-2k\Phi_{1I}\left(z_{D}\right)+2a_{1R}\left(z_{D}\right)\right]$$

where the intensity of undisturbed PB is expressed by:

(10) $$I_{0g}\left(x_{D},y_{D},z_{D}\right)=A_{0}{}^{2}\left(z_{D}\right){\left|\exp\left[ik\Phi_{0}\left(z_{D}\right)\right]\right|}^{2}$$

whereas the phase of undisturbed PB is:

(11) $$\Phi_{0}\left(z_{D}\right)=n_{0}z_{D}\left(1-\frac{\xi_{0}^{2}+\eta_{0}^{2}}{2z_{RC}^{2}}\right)$$

and undisturbed PB’s ray in the input plane of the experimental setup:

(12) $$\xi_{0}=x_{D}{\left(1+i\frac{z_{D}}{z_{RC}}\right)}^{-1},\;\xi_{0}=y_{D}{\left(1+i\frac{z_{D}}{z_{RC}}\right)}^{-1}$$

The obtained photodeflection (PD) signal consists of two components that, in case of detection by the use of a quadrant photodiode (QP), are given by:

(13) $$S_{PDn}=K_{d}\left(\overset{+\infty }{\underset{0}{\int }}-\overset{0}{\underset{-\infty }{\int }}\;\right)dz_{D}\overset{+\infty }{\underset{-h}{\int }}dy_{D}I\left(x_{D},y_{D},z_{D}\right)$$

(14) $$S_{PDt}=K_{d}\overset{+\infty }{\underset{-h}{\int }}dx_{D}\left(\overset{+\infty }{\underset{0}{\int }}-\overset{0}{\underset{-\infty }{\int }}\;\right)dy_{D}I\left(x_{D},y_{D},z_{D}\right)$$

Here, *K_d_* is the detector’s constant, *h* is the height of PB over the sample, *S_PDn_* is the normal component of the PD signal being a consequence of the difference in illumination between upper and lower photodiode’s halves and *S_PDt_* is the tangential component of the PD signal resulting from a difference in illumination between left and right photodiode’s halves. It is taken into account in Equations (13) and (14) that the detector is partly covered by the sample.

The in-plane thermal diffusivities and conductivities of GNP samples were found by collecting the *S_PDt_* signal as a function of the distance between the EB and PB and performing the multiparameter fitting of curves obtained from Equation (14) to the experimental data.

### 2.2. Slope Method

**Figure 1:**
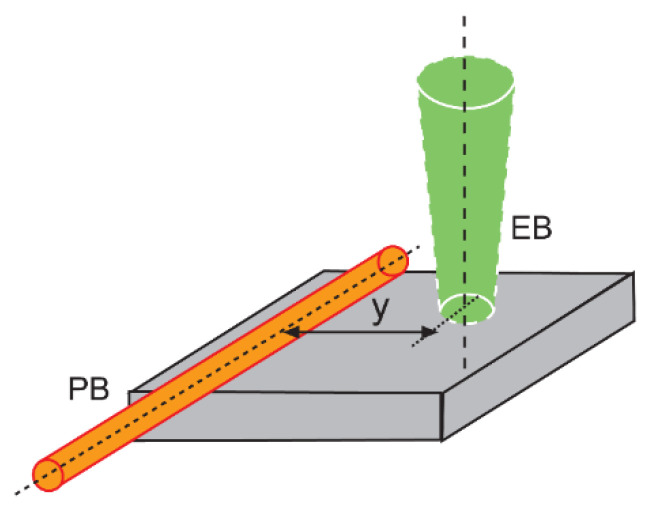
Schematic diagram of EB and PB configuration in the in-plane thermal diffusivity determination by the slope method.

The GNP samples in-plane thermal diffusivities were verified by determining the slope of the tangential component of the PBDS signal phase dependence *θ* from the position-sensitive detector on a distance y between the pump and probe beam axes (Figure 1):

(15) θ=ay+b

where *b* is the intercept determined by the geometry of the experimental setup (such as the height of PB over the sample, detector and sample position, radius of PB and its waist position, etc.). The slope *a* is found by the use of expression [25]:

(16) $$a=-\sqrt{\frac{\pi f}{D_{T-in}}}$$

where *f* is the modulation frequency of EB and *D_T-in_* is the in-plane thermal diffusivity of GNP material.

The uncertainty of *D_T-in_* determination was found by the use of an error propagation algorithm and had the form:

(17) $$∆D_{T-in}=2\pi f\frac{∆a}{a^{3}}$$

where Δ*a* is the standard deviation of the regression curve slope.

### 2.3. Frequency Scan Method

The through-plane thermal diffusivity and conductivity of GNP samples were found by the frequency scan method in which the amplitude and phase of the *S_PDn_* signal are collected as a function of the modulation frequency of TOs. To the amplitude and phase of experimental data, the theoretical curves are fitted by the use of the least-squares fitting procedure.

**Figure 2:**
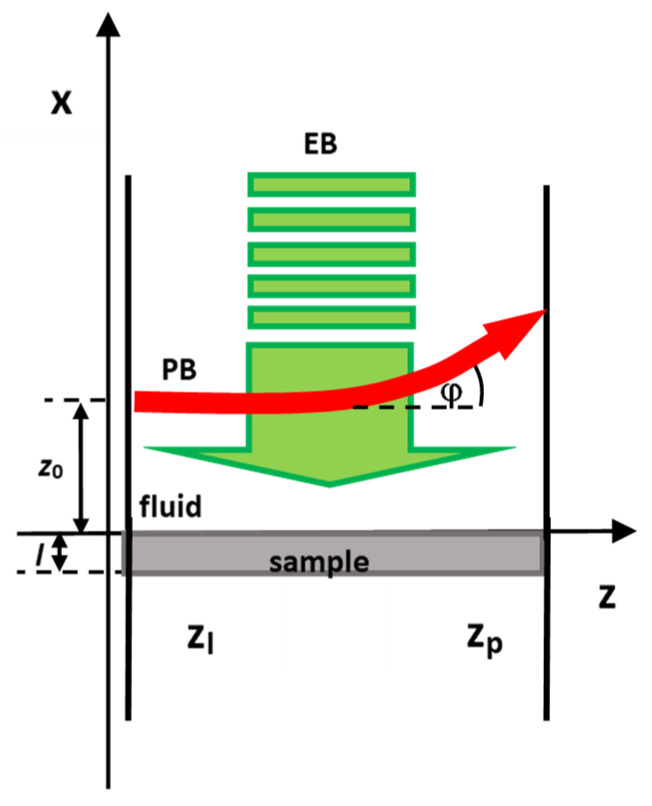
Geometry of the PBDS experiment.

If we defocus EB so that it is much wider than PB, the induced TOs are 1D (Figure 2):

(18) $$\upsilon_{f}=b_{f}\cos\left(\Omega t+\varphi_{f}\right)$$

where *b_f_* is the amplitude of TOs at sample’s surface, *φ_f_* is the phase shift between the phase of sample surface’s temperature change and the phase of the pump beam, *Ω* is the angular modulation frequency of the EB and t is the time. Both *b_f_* and *φ_f_* are functions of the sample’s thermal (thermal diffusivity and conductivity) and geometrical properties (thickness), as well as parameters of the experimental setup (height of PB over the sample’s surface) [24].

TOs change only the coordinate of the PB trajectory in the direction of TOs propagation (*x*). The proper correction to PB trajectory is then expressed by Equation (4). After integration, we obtained:

(19) $$x_{1}=\sqrt{2}\left(z-z_{s}\right)\left(z_{p}-z_{l}\right)b_{f}k_{ft}s_{T}\exp\left[-\Omega^{\frac{1}{2}}{\left(2D_{f}\right)}^{-\frac{1}{2}}z_{0}\left(\tau_{s}\right)\right]\times \sin\left[\Omega t-\Omega^{\frac{1}{2}}{\left(2D_{f}\right)}^{-\frac{1}{2}}z_{0}\left(\tau_{s}\right)+\varphi_{f}-\pi /4\right]$$

(20) $$z_{0}\left(\tau_{s}\right)=\xi \left(1+iz_{RC}^{-1}\tau_{s}n_{0}\right),\;\tau_{s}=\left(z_{p}+z_{l}\right){\left(2n_{0}\right)}^{-1}$$

where *D_f_* is the thermal diffusivity of the fluid above the sample; *z_l_* and *z_p_* are the positions of the left and right edges of the medium where step change occurs; *k_ft_* is the wavenumber of TOs. 

The correction to the amplitude (*A*) of the PB electric field at the detector (*z_D_*) then has the form:

(21) $$a_{1d}=-{\left(2n_{0}^{2}\right)}^{-1}\left(z_{D}-z_{s}\right)\left(z_{p}-z_{l}\right)\frac{\partial P}{\partial \xi }{\left(1+iz_{D}z_{RC}{}^{-1}\right)}^{-1}$$

and

(22) $$P\left(\xi \right)=\sqrt{2}n_{0}^{2}b_{f}k_{ft}s_{T}\exp\left[-\Omega^{\frac{1}{2}}{\left(2D_{f}\right)}^{-\frac{1}{2}}z_{0}\left(\tau_{s}\right)\right]\sin\left[\Omega t-\Omega^{\frac{1}{2}}{\left(2D_{f}\right)}^{-\frac{1}{2}}z_{0}\left(\tau_{s}\right)+\varphi_{f}-\pi /4\right]$$

and both correction *ψ*_1*d*_ and *ψ*_1*f*_ corrections to PB phase at the detector (*z_D_*) are:

(23) $$\psi \left(z_{D}\right)=\psi_{0}\left(z_{D}\right)+n_{0}^{2}s_{T}\overset{\tau }{\underset{0}{\int }}\upsilon_{f}\left(x,y\right)d\tau^{\prime }=\psi_{0}\left(z_{D}\right)+\psi_{1d}\left(z_{D}\right)+\psi_{1f}\left(z_{D}\right)$$

where *ψ*_0_ is the phase of undisturbed PB. The *ψ*_1*d*_ and *ψ*_1*f*_ corrections to the PB phase are expressed by:

(24) $$\psi_{1d}\left(z_{D}\right)=z_{D}{\left(n_{0}z_{RC}^{2}\right)}^{-1}\left(z_{D}-z_{s}\right)\left(z_{p}-z_{l}\right)P\left(\xi_{D0}\tau_{s0}\right){\left(1+iz_{D}z_{RC}{}^{-1}\right)}^{-2}$$

(25) $$\psi_{1f}\left(z_{D}\right)=n_{0}s_{T}b_{f}\left(z_{p}-z_{l}\right)\exp\left[-k_{ft}x\left(\xi_{D0},\tau_{s0}\right)\right]\cos\left[\Omega t-k_{ft}z\left(\xi_{D0},\tau_{s0}\right)+\varphi_{f}\right]$$

where *ξ*_*D*0_ and *τ*_*s*0_ are the coordinate of the ray in the input plane of the experimental setup for the given observation point and the running coordinate along the ray over the sample for undisturbed probe beam, respectively.

Based on the above equations, the photodeflection signal (PDS) can be written as:

(26) SPDn=2Kd(∫0+∞−∫−h0 )dz∫−∞+∞dx[Re(a1d)−Im(ψ1d+ψ1f)]I0g=SPDnd+SPDnf=Atcos(Ωt+φt)

where *K_d_* is the detector constant, *I*_0*g*_ is the light intensity of undisturbed PB, *S_PDnd_* and *S_PDnf_* are the components of PDS resulting from deflection of PB in the field of TOs and its phase change, and *A_t_* and *φ*_t_ are the amplitude and additional phase changes in total PDS.

### 2.4. Fitting Accuracy

The fitting accuracy of the determined parameter (*S_Ds_*) was estimated in both surface and frequency scan methods by calculating the square root of the covariance matrix [26]:

(27) $$S_{p}=\sqrt{cov\left(P\right)}$$

where *P* is the fitted parameters matrix

(28) $$cov\left(P\right)=\sigma_{r}{\left(J_{f}^{T}J_{f}\right)}^{-1}$$

and *σ_r_* is the variance on residuals

(29) $$\sigma_{r}=\frac{1}{N-k}\overset{N}{\underset{i=1}{\sum }}{\left[y_{i}-f\left(P^{\prime }\right)\right]}^{2}$$

where *N* is the number of points in the dataset and *J_f_* denotes the Jacobian matrix; meanwhile, *P*′ is the matrix of fitted parameters for which the minimum error function was reached.

## 3. Materials and Methods

### 3.1. Sample Preparation

The GNP samples were prepared by filtration in the form of sheets. Unfortunately, they were brittle and easy to break, which makes them difficult to be measured directly. Thus, they were compressed by the use of an MTS loading machine equipped with a load cell MTS 661.20F-02 (MTS systems corporation, Eden Prairie, MN, USA). The examined samples were pressed with loads from 500 to 2000 N. The complete procedure about the preparation process was already discussed elsewhere [12,13]. Prepared samples with different loads pressures were denominated as follows: S1: GPN1 (500 N), S2:GPN2 (1000 N), S3: GPN3 (2000 N), S4: INFN (700 N), S5: NANESA (700 N), S6: Graphene UP (700 N), S7: Graphene UP1 (500 N), S8: Graphene UP2 (1000 N), S9: Graphene UP3 (2000 N), S10: GPN non-pressed (0 N).

Different pressure loads were performed to obtain a sizeable density of samples, which can be higher than the non-pressed sample. Therefore, it is possible to test the influence of the increase in density on the heat transmission properties of the samples. The values of the density for the samples pressed at increasing load can be found in Table 1 of references [12,13]; hence, we can estimate a range of density values between 350 and 650 Kg/m^3^.

### 3.2. Experimental Setup

**Figure 3:**
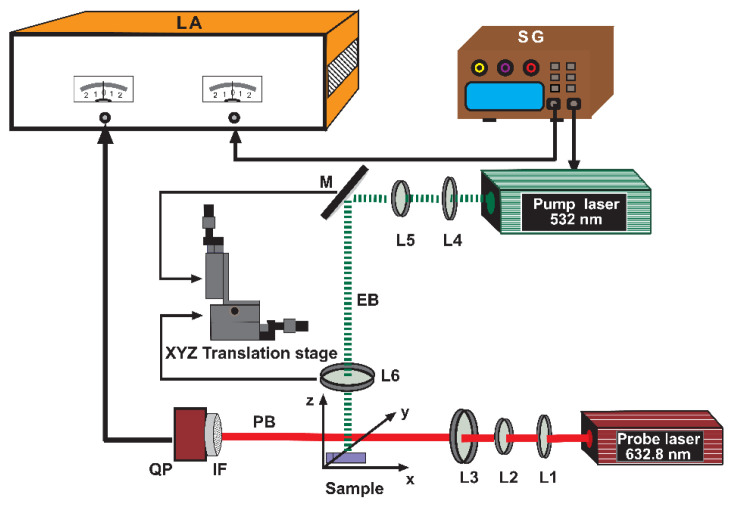
Scheme of the experimental setup for BDS measurement. L1, L2, L3, L4, L5, L6: lenses, M: reflecting mirrors, QP: quadrant photodetector, IF: filter, LA: lock-in amplifier, EB: excitation beam, PB: probe beam, SG: signal generator.

The thermal diffusivities of GNP samples after pressing with different loads were determined by the use of the slope beam deflection method [22] and the experimental setup shown in Figure 3. The source of the electromagnetic radiation (excitation beam EB) is the 532 nm diode-pumped solid-state laser (MGL-III-532-100, Ultra Lasers, Newmarket, ON, Canada) modulated at 11 Hz using a signal generator (RIGOL DG1022, RIGOL Technologies, Inc., Portland, OR, USA). It is directed perpendicularly by a mirror (M) onto the sample surface and focused to a spot of about 50 µm by a set of three lenses L4, L5, L6 with 40 mm, 100 mm, 150 mm focal lengths (LB1757-A, LB1676-A, LB1757-A, Thorlabs, Newton, NJ, USA), respectively. The lens L6 and the mirror M were placed on an XYZ translation stage to move the excitation beam in the y-direction with a 12.42 µm step. The EB absorption induces temperature oscillations (TOs) in the sample and in the surrounding air, which are further detected by a laser probe beam (PB) (He-Ne, 3 mW, 05-UR-111, Melles Griot, Carlsbad, CA, USA) of 632.8 nm wavelength and 2 mW output power that passes the sample adjacent medium skimming its surface.

The probe beam was also collimated and focused into a spot of 50 µm diameter over the sample by a set of lenses L1, L2, L3 with 40 mm, 100 mm, 150 mm focal lengths (LB1757-A, LB1676-A, LB1757-A, Thorlabs), respectively. The TOs produce changes in the air refractive index and its gradients that in turn cause the intensity change in PB measured by a position-sensitive detector (QP) (PDQ80A, Thorlabs) equipped with an interference filter (IF) (632.8 nm CWL, Thorlabs) and connected to the lock-in amplifier (Stanford Research System, Model SR5 10, Sunnyvale, CA, USA) as well as PC for data acquisition and processing. In the case of EB, it enables collecting the signal as a function of a distance y between the EB and PB axes, thus performing the slope method measurements. As a result of putting the GNP sample on a 3D translation stage, it is possible to optimize the experimental configuration [27]. All measurements were performed in air at room temperature.

Furthermore, in the case of the slope method, EB was tightly focused at 50 µm, whereas for the frequency scan method, it was defocused at 200 µm to ensure the 1D geometry of the configuration.

## 4. Results and Discussion

### 4.1. Surface Scan Method

**Figure 4:**
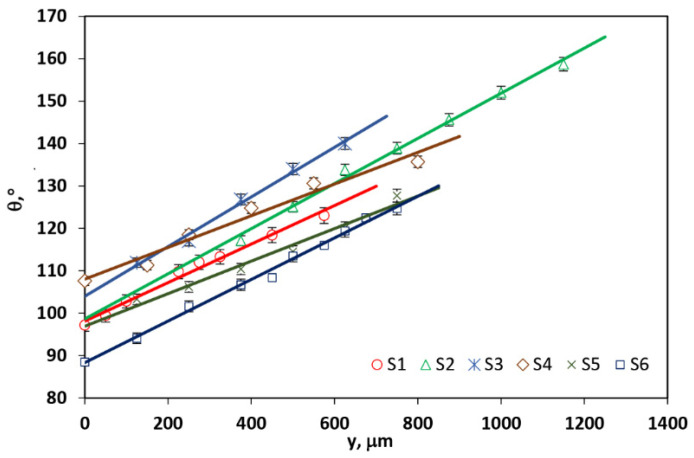
Variation in the photothermal deflection phase with pump-probe beam offset for GNP samples pressed with different loads.

Figure 4 presents the phase of the *S_PDt_* tangential component of the signal dependence on the excitation-probe beam offset *y*. It is observed that the phase of *S_PDt_* changes linearly with the increase in the value of *y*. The results of the least-squares fitting procedure of the *S_PDt_* phase of experimental data to the theoretical curves obtained by the use of Equation (14) are presented in Table 1.

The validity of the fitting procedure was tested by simulating data sets and performing the fitting with different initial values of unknown variables, and studying whether the obtained values of fitted parameters make physical sense or not. The obtained results were then verified by the slope method.

### 4.2. Slope Method

Equation (15) can be used in the case of large EB-PB offsets y compared to EB spot size [25]. This condition is satisfied since the EB has a spot of 50 µm, whereas the distance *y* between the EB and PB axes is changed in the range from 0 to about 1 mm by a step of 12.42 µm. The linear relation between the phase of PBDS tangential component of the signal and the EB-PB offsets y is valid only in case of infinitely thin both EB and PB, as well as PB skimming the sample surface (its height over the sample should be close to zero) [25,28,29,30]. For wider EB and PB, the nonlinearities on the *θ*(*x*) dependence occur, which are bigger as much wider both beams are. Furthermore, the characteristic becomes shifted by a constant value. Moreover, its slope is changed for high modulation frequencies of TOs, which is masked by performing the measurement in the frequencies range between 1 and 11 Hz.

It must also be stated that the *θ*(*x*) relation holds the linear dependence for the GNPs grain size *L_GS_* small compared to the spatial range of the thermal disturbance *L_TH_* that is defined as [25,28,29,30]:

(30) $$L_{TH}\approx 2\pi \mu \;$$

where thermal diffusion length *µ* can be found by the of equation:

(31) $$\mu =\sqrt{\frac{D_{T-in}}{\pi f}}$$

The modulation frequencies used in our experiments were 11 Hz and gave the values of *L_th_* higher than 5 mm for the lowest obtained values of *D_T-in_* and highest frequency used. Since the examined samples are nanoplatelets, the condition of *L_CH_* ≪ *L_TH_* is satisfied, and the induced TOs are not affected by the material’s structure. Therefore, the measured thermal diffusivity is a bulk effective diffusivity of the examined material, and Equation (16) can be used for its determination. The values of in-plane thermal diffusivities were measured as an average of three different measurements on different spots on the GNP samples’ surface for which *SD* of *D_T-in_* was determined to evaluate the determination repeatability.

The parameters of the linear dependences of the tangential component of the PDS phase on the EB-PB offsets, presented in Figure 4, are shown in Table 2.

The values of the GNP samples in-plane thermal diffusivities are evaluated from the slope of the plots according to Equation (16). Their average values and standard deviations are presented in Table 3.

It is seen from Table 1 and Table 2 that in-plane thermal diffusivity decreases with an increase in the pressing loads, which is opposite to the expected effect. The reason for that could be folding, rolling or breaking of platelets creating the GNP samples caused by pressing loads and thus introducing more interfaces that increase the thermal resistance of the material preventing the heat conduction and its exchange with the surroundings. By comparing the data obtained by surface scan and slope method (Table 1 and Table 3), no significant differences were found at a 95% confidence interval. The calculated p-value is smaller than 0.05 for all analyzed samples. These results demonstrated the reliability of the obtained results as well as the agreement between the developed theory and the experimental values.

### 4.3. Frequency Scan Method

**Figure 5:**
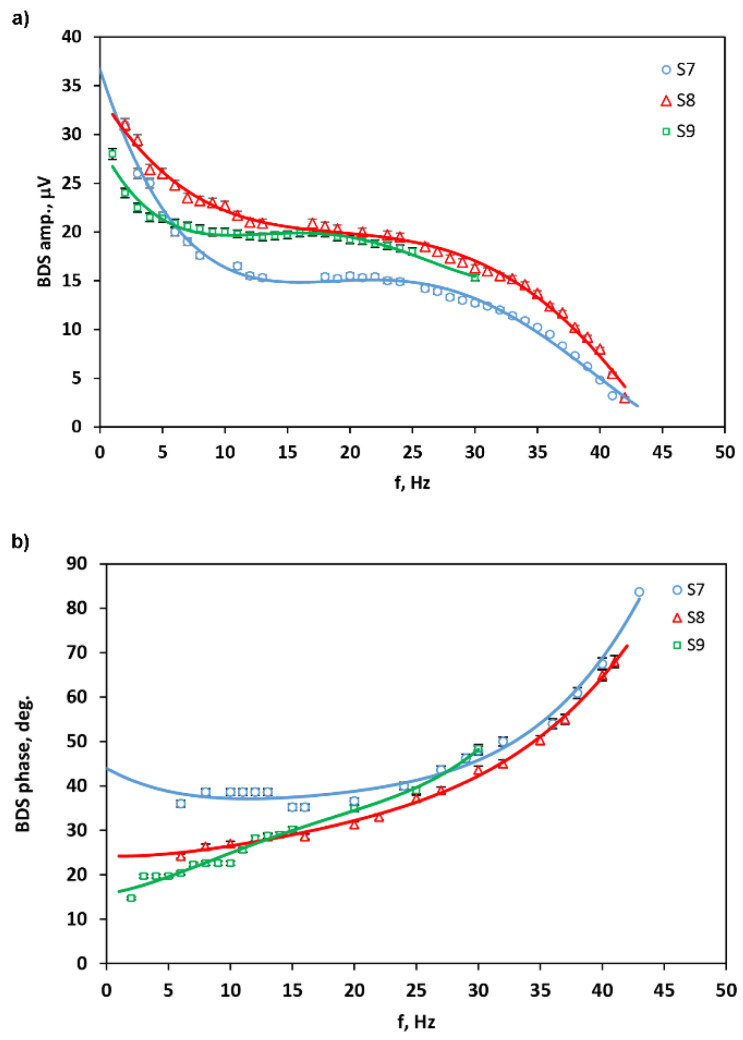
The amplitude (**a**) and phase (**b**) of PBDS signal dependence on modulation frequency of EB.

Figure 5 presents the example of amplitude and phase of the BDS signal dependences on the modulation frequency of the excitation beam for GNP (S7–S9) samples pressed by different loads. The theoretical curves received by the use of CRT are fitted to the experimental data [31]. The fitted parameter was the through-plane thermal diffusivity and conductivity of GNP samples. It is seen that the BDS signal decreases rapidly with the increase in EB modulation frequency, which is the consequence of shortening the thermal diffusion length of TOs, preventing the heat from reaching PB and being fully distorted. Thus, the experimental setup was optimized by tightly focusing PB and aligning it close to the sample surface. Furthermore, the used samples were flatted and of small lateral dimensions.

The validity of the fitting procedure was tested in the same way as in the case of the slope method. The results are presented in Table 4.

It is expected that thermal diffusivity increases with the increase in the sample pressing loads. However, the data presented in Table 4 for samples S0–S6 and S7–S9 reveal a decreasing trend in the values of GNP thermal diffusivity with load application. The GNP samples prepared by pressing in the laboratory are a mixture of solid graphene nanoplatelets and air. Their compression causes a reduction in the air content among solid platelets and increases in the number of contact points between single nanoplatelets, which should increase the value of the material’s thermal diffusivity. On the other side, the load application results in GNP samples folding, rolling and breaking, which introduces additional interfaces between platelets and thus, increase the material thermal resistance decreasing its value of thermal diffusivity. These two effects compete, causing the second to have a dominating effect on the values of GNP thermal diffusivities.

### 4.4. Comparison of In-Plane and Through-Plane Thermal Properties

Both the values of in-plane thermal diffusivities and conductivities are about five times higher than those of through-plane ones. The explanation of that is the way of samples preparation and consequently their structure. The pressing loads are applied in the direction perpendicular to the sample samples’ surface, compressing them in this direction, due to which the reduction in the through-plane values are observed compared to the values of pure graphene (3000 Wm^−1^K^−1^). This is the result of breaking the GNP platelets in this direction, which introduces additional interfaces and thus, limits the heat conduction within the samples and its exchange with surroundings. Since no pressing loads are applied in the direction parallel to the sample’s surface, the resulting change in their structure (folding, rolling, breaking platelets) in this direction exceeds much less extends and results in much higher in-plane values of thermal properties.

### 4.5. Thermal Conductivity Evaluation

**Figure 6:**
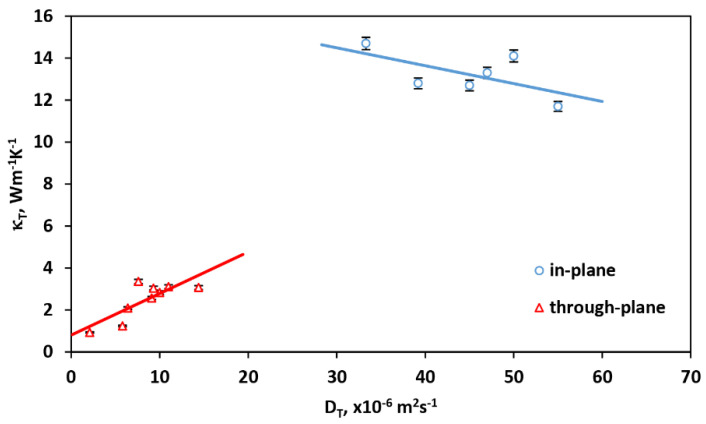
The relation between thermal diffusivity and conductivity for in-plane and through-plane configuration of the measurements.

The GNPs values of both through-plane and in-plane thermal conductivity were verified, as well as the uncertainty of their determination was obtained by the use of equations:

(32) $$\kappa_{T}=D_{T}c_{p}\rho$$

(33) $$∆\kappa_{T}=c_{p}\sqrt{{\left(\rho \Delta D_{T}\right)}^{2}+{\left(D_{T}\Delta \rho \right)}^{2}}$$

where *c_p_* is the material-specific heat and *ρ* its density. The specific heat of GNPs was assumed to be 710 Jkg^−1^K^−1^ [32], whereas their densities as described in [12,13]. The results are presented in Table 5 and Figure 6.

It was observed that there is a linear dependence between the thermal diffusivity and conductivity for both in-plane and through-plane thermal properties.

In the in-plane case, the thermal conductivity decreases with the increase in thermal diffusivity, whereas *K_T-through_* increases with the increase in *D_T-through_* for the whole range of the loading pressure used. Such behavior is caused by different heat propagation in relation to the direction of load application, as a result of different changes introduced in the GNP samples structure after their compression, as described above.

## 5. Conclusions

In this work, both the in-plane and through-plane thermal diffusivities and conductivities of GNP material were determined by the use of photothermal beam deflection spectrometry, for which a new theoretical description was developed. The samples were prepared in the form of sheets on which different pressing loads were applied. As a result, the obtained through-plane values of thermal properties were around five times lower than those in-plane. Furthermore, the thermal properties in the direction parallel and perpendicular to the sample surface behaved differently because of different GNPs structures in these directions, which causes different heat conduction and its exchange with the surrounding. By taking into account the many possible practical application of graphene, the knowledge of its properties is crucial to predict its behavior in industrial applications. Among the many exceptional properties of graphene (mechanical, electrical, electronic, chemical, etc.), the thermal and thermophysical properties have a large impact in concrete applications related to heat-conducting systems, as well as heat storage systems. From the results obtained in the present paper, it can be concluded that the GNP material’s density (adjusted by application of different loads) is a crucial factor that strongly influences the material thermal parameters and further determines their possible commercial application in different fields of industry and medical science, etc. The through-plane/in-plane measurements of GNP samples require careful alignment of BDS experimental setup and consideration of factors such as the height of the PB over the sample, its position relative to EB and the modulation frequency of TOs, etc., since they cannot be eliminated, due to the limitations of the BDS apparatus.

## Figures and Tables

**Table 1:** Values of the GNP samples in-plane thermal diffusivities and conductivities pressed with loads from 500 to 2000 N obtained by the use of CRT theory.

Sample	*P*, N	*D_T-in_*, ×10^−6^ m^2^s^−^^1^	*κ_T-in_*, W m^−1^K^−1^
S1	500	46.0 ± 2.2	10.2 ± 0.4
S2	1000	34.4 ± 1.4	10.8 ± 0.4
S3	2000	30.2 ± 0.8	13.4 ± 0.6
S4	700	41.5 ± 1.4	11.2 ± 0.6
S5	700	45.0 ± 1.5	12.7 ± 0.7
S6	700	42.6 ± 1.1	11.9 ± 0.8
S10	0	107.0 ± 2.0	24.5 ± 1.8

**Table 2:** Parameters of the *θ*(*x*) linear dependences.

Sample	*a*, m^−1^	*b*, °	*R* ^2^
S1	0.0454	98.13	0.9868
S2	0.0532	98.66	0.9943
S3	0.0586	103.98	0.9915
S4	0.0374	108.03	0.968
S5	0.0384	96.92	0.9742
S6	0.0490	88.33	0.9944

**Table 3:** Values of the GNP samples in-plane thermal diffusivities pressed with loads from 500 to 2000 N.

Sample	*P*, N	*f*, Hz	*D_T-in_*, ×10^−6^ m^2^s^−1^
S1	500	11	50.0 ± 3.2
S2	1000	11	39.2 ± 1.6
S3	2000	11	33.3 ± 1.1
S4	700	11	45.0 ± 1.8
S5	700	11	50.0 ± 2.0
S6	700	11	47.0 ± 1.7
S10	0	11	110 ± 2.4

**Table 4:** Values of the GNP samples through-plane thermal diffusivities pressed with loads from 500 to 2000 N.

Sample	*P*, *N*	*D_T-through_*, ×10^−6^ m^2^s^−1^	*κ_T-through_*, W m^−1^K^−1^
S1	500	14.4 ± 0.2	3.23 ± 0.10
S2	1000	9.30 ± 0.11	3.16 ± 0.08
S3	2000	7.60 ± 0.08	3.52 ± 0.12
S4	700	11.0 ± 0.1	3.28 ± 0.14
S5	700	10.0 ± 0.1	3.11 ± 0.12
S6	700	9.10 ± 0.11	2.74 ± 0.10
S7	500	5.80 ± 0.06	1.33 ± 0.06
S8	1000	6.40 ± 0.08	2.25 ± 0.08
S9	2000	2.10 ± 0.02	1.08 ± 0.04
S10	0	99.5 ± 1.8	

**Table 5:** Values of the GNP samples through-plane and in-plane thermal conductivities pressed with loads from 500 to 2000 N.

Sample	*ρ*, kg m^−3^	*κ_T-in_*, W m^−1^K^−1^	*κ_T-through_*, W m^−1^K^−1^
S1	300 ± 7	11.7 ± 0.6	3.07 ± 0.12
S2	461 ± 9	12.8 ± 0.7	3.04 ± 0.10
S3	623 ± 18	14.7 ± 0.7	3.36 ± 0.16
S4	398 ± 8	12.7 ± 0.6	3.11 ± 0.11
S5	398 ± 8	14.1 ± 0.8	2.83 ± 0.09
S6	398 ± 8	13.3 ± 0.7	2.57 ± 0.10
S7	300 ± 7	-	1.24 ± 0.04
S8	461 ± 9	-	2.09 ± 0.06
S9	623 ± 18	-	0.93 ± 0.03

## Data Availability

The data presented in this study are available on request from the corresponding author.

## Appendix A

The complex form of the temperature distribution in the fluid above the GNP sample surface is given by:

(A1) $$\begin{array}{l} \upsilon_{f}\left(x,y\right)=\gamma_{0}\{\pi \exp\left[-x\sqrt{i\omega /D_{Tf}}\right]\left(\mathrm{erf}\left[{\left(2x\sqrt{i\omega /D_{Tf}}\right)}^{-1/2}\left(y+a\right)\right]-\mathrm{erf}\left[{\left(2x\sqrt{i\omega /D_{Tf}}\right)}^{-1/2}\left(y-a\right)\right]\right)\\ \;+\pi \exp\left[-x\sqrt{i\omega /D_{Tf}}+2d\sqrt{i\omega /D_{Tm}}\right]\mathrm{erf}\left[{\left(2\right)}^{-1/2}{\left(x\sqrt{D_{Tf}/i\omega }+2d\sqrt{D_{Tm}/i\omega }\right)}^{-1/2}\left(y-a\right)\right]\\ \;-\mathrm{erf}\left[{\left(2\right)}^{-1/2}{\left(x\sqrt{D_{Tf}/i\omega }+2d\sqrt{D_{Tm}/i\omega }\right)}^{-1/2}\left(y+a\right)\right]\} \end{array}$$

(A2) $$\gamma_{0}=-{\left[2\pi /q_{0}\left(\sqrt{i\omega /D_{Tf}}k_{Tf}+\sqrt{i\omega /D_{Tm}}k_{Tm}\right)\right]}^{-1}$$

where *q*_0_ is the average energy flux that impinges on the illuminated sample’s surface, *d* is the sample’s thickness, *a* is the radius of PB in its waist, *D_Tm_, D_Tf_, k_Tf_, k_Tm_* are the GNP material and fluid over its surface thermal diffusivities and conductivities, respectively.

## Appendix B

After calculating the proper integrals in Equations (4) and (5), the correction to coordinates *y* and *z* of PB trajectory has forms:

(A3) $$x_{1}\left(\xi ,\eta ,\tau \right)=\left(\tau -\tau_{s}\right)\left(\tau_{p}-\tau_{l}\right)P\left(\xi ,\eta \right)$$

(A4) $$\begin{array}{l} P\left(\xi ,\eta \right)=\pi n_{0}^{2}s_{T}\gamma_{0}\{\exp\left(-k_{gI}x_{0s}\right)[-k_{gI}\left(\mathrm{erf}\left[\sqrt{k_{gI}/2x_{0s}}\left(y_{0s}+a\right)\right]-\mathrm{erf}\left[\sqrt{k_{gI}/2x_{0s}}\left(y_{0s}-a\right)\right]\right)\\ \;+\sqrt{k_{gI}/2\pi }x_{0s}{}^{-3/2}\left(-\left(y_{0s}+a\right)\exp\left[-k_{gI}/2x_{0s}{\left(y_{0s}+a\right)}^{2}\right]+\left(y_{0s}-a\right)\exp\left[-k_{gI}/2x_{0s}{\left(y_{0s}-a\right)}^{2}\right]\right)]\\ \;+\exp(-k_{gI}x_{0s}\\ \;+2dk_{sI})[-k_{gI}\left(\mathrm{erf}\left[\sqrt{{\left(2x_{0s}/k_{gI}+2d/k_{sI}\right)}^{-1}}\left(y_{0s}-a\right)\right]-\mathrm{erf}\left[\sqrt{{\left(2x_{0s}/k_{gI}+2d/k_{sI}\right)}^{-1}}\left(y_{0s}+a\right)\right]\right)\\ \;+{\left(k_{gI}\sqrt{2\pi }\right)}^{-1}{\left(x_{0s}/k_{gI}+2d/k_{sI}\right)}^{-3/2}(-\left(y_{0s}+a\right)\exp\left[-{\left(x_{0s}/k_{gI}+2d/k_{sI}\right)}^{-1}{\left(y_{0s}+a\right)}^{2}/2\right]\\ \;+\left(y_{0s}-a\right)\exp\left[-{\left(x_{0s}/k_{gI}+2d/k_{sI}\right)}^{-1}{\left(y_{0s}-a\right)}^{2}/2\right])]\}\exp\left(i\omega t\right) \end{array}$$

(A5) $$y_{1}\left(\xi ,\eta ,\tau \right)=\left(\tau -\tau_{s}\right)\left(\tau_{p}-\tau_{l}\right)G\left(\xi ,\eta \right)$$

(A6) $$\begin{array}{l} G\left(\xi ,\eta \right)=2\sqrt{\pi }n_{0}^{2}s_{T}\gamma_{0}\{\sqrt{k_{gI}/2x_{s}}\exp\left(-k_{gI}x_{0s}\right)\left(\exp\left[-k_{gI}/2x_{0s}{\left(y_{0s}+a\right)}^{2}\right]-\exp\left[-k_{gI}/2x_{0s}{\left(y_{0s}-a\right)}^{2}\right]\right)\\ \;+{\left(2x_{0s}/k_{gI}+4d/k_{sI}\right)}^{-1/2}\exp\left(-k_{gI}x_{0s}-2dk_{sI}\right)(\exp\left[-{\left(2x_{0s}/k_{gI}+4d/k_{sI}\right)}^{-1}{\left(y_{0s}-a\right)}^{2}\right]\\ \;-\exp\left[-{\left(2x_{0s}/k_{gI}+4d/k_{sI}\right)}^{-1}{\left(y_{0s}+a\right)}^{2}\right])\}\exp\left(i\omega t\right) \end{array}$$

(A7) $$\tau_{s}=\left(z_{p}+z_{l}\right)/2n_{0}$$

(A8) $$x_{0s}=\xi \left(1+in_{0}\tau_{s}/z_{RC}\right),\;y_{0s}=\eta \left(1+in_{0}\tau_{s}/z_{RC}\right)$$

## Appendix C

The correction to the PB phase is created by the change in its optical path produced by the change in air refractive index and its gradients; thus, it consists of two components:

(A9) $$\Phi_{1}=\Phi_{1f}+\Phi_{1d}$$

where *Φ*_1*f*_ results from the PB phase change caused by the change in fluid refractive index, *Φ*_1*d*_ is the consequence of existence of fluid refractive index gradients that lead to the change in PB geometrical path. The proper correction is given by:

(A10) $$\begin{array}{l} \Phi_{1f}=\pi n_{0}^{2}s_{T}\gamma_{0}\left(\tau_{p}-\tau_{l}\right)\{\exp\left(-k_{gI}x_{s}\right)\left(\mathrm{erf}\left[\sqrt{k_{gI}/2x_{s}}\left(y_{s}+a\right)\right]-\mathrm{erf}\left[\sqrt{k_{gI}/2x_{s}}\left(y_{s}-a\right)\right]\right)\\ \;+\exp\left(-k_{gI}x_{s}-2dk_{sI}\right)\mathrm{erf}\left[\sqrt{{\left(2x_{s}/k_{gI}+4d/k_{sI}\right)}^{-1}}\left(y_{s}-a\right)\right]\\ \;-\mathrm{erf}\left[\sqrt{{\left(2x_{s}/k_{gI}+4d/k_{sI}\right)}^{-1}}\left(y_{s}+a\right)\right]\}\exp\left(i\omega t\right) \end{array}$$

(A11) $$x_{0s}=x\left(\xi ,\tau_{s}\right),\;y_{0s}=y\left(\eta ,\tau_{s}\right)$$

(A12) $$x_{s}=x_{0s}+x_{1s},\;y_{s}=y_{0s}+y_{1s}$$

(A13) $$x_{1s}=-\left(\tau_{D}-\tau_{s}\right)\left(\tau_{p}-\tau_{l}\right)P\left(\xi_{0},\eta_{0}\right){\left(1+iz_{D}/z_{RC}\right)}^{-1}\left(1+in_{0}\tau_{s}/z_{RC}\right)$$

(A14) $$y_{1s}=-\left(\tau_{D}-\tau_{s}\right)\left(\tau_{p}-\tau_{l}\right)G\left(\xi_{0},\eta_{0}\right){\left(1+iz_{D}/z_{RC}\right)}^{-1}\left(1+in_{0}\tau_{s}/z_{RC}\right)$$

(A15) $$\xi_{0}=x_{D}{\left(1+iz_{D}/z_{RC}\right)}^{-1},\;\eta_{0}=y_{D}{\left(1+iz_{D}/z_{RC}\right)}^{-1}$$

(A16) $$\tau_{D}=z_{D}/z_{RC}^{2}n_{0}\left\{\left[1-\left(\xi_{0}^{2}+\eta_{0}^{2}\right)/2\right]-\left(\xi_{0}\xi_{1}+\eta_{0}\eta_{1}\right)\right\}$$

(A17) $$\xi_{1}=-\left(\tau_{D}-\tau_{s}\right)\left(\tau_{p}-\tau_{l}\right)P\left(\xi_{0},\eta_{0}\right){\left(1+iz_{D}/z_{RC}\right)}^{-1}$$

(A18) $$\eta_{1}=-\left(\tau_{D}-\tau_{s}\right)\left(\tau_{p}-\tau_{l}\right)G\left(\xi_{0},\eta_{0}\right){\left(1+iz_{D}/z_{RC}\right)}^{-1}$$

(A19) kg=ω/iDTf, kgI=Im(ω/iDTf)

(A20) ks=ω/iDTs, ksI=Im(ω/iDTs)

(A21) $$\Phi_{1d}=z_{D}/z_{RC}^{2}n_{0}\left(\tau_{D}-\tau_{s}\right)\left(\tau_{p}-\tau_{l}\right){\left(1+iz_{D}/z_{RC}\right)}^{-2}\left[x_{D}P\left(\xi_{0},\eta_{0}\right)+y_{D}G\left(\xi_{0},\eta_{0}\right)\right]\exp\left(i\omega t\right)$$

*x_D_*, *y_D_*, *z_D_* are the coordinates of PB’s rays in the detector plane.

## Appendix D

The correction to the PB amplitude at the detector (yD) is expressed by:

(A22) $$a_{1}\left(z_{D}\right)=-\frac{z_{D}\left(z_{p}-z_{l}\right)}{2n_{0}^{2}}\frac{1+iz_{s}/z_{RC}}{1+iz_{D}/z_{RC}}\left({\frac{\partial P}{\partial \xi }|}_{\begin{array}{c} \xi =\xi_{0}\\ \eta =\eta_{0} \end{array}}+{\frac{\partial G}{\partial \eta }|}_{\begin{array}{c} \xi =\xi_{0}\\ \eta =\eta_{0} \end{array}}\right)$$

(A23) $$z_{s}=\left(z_{p}+z_{l}\right)/2$$
